# Intrauterine transfusion under fetal analgesia: the evaluation of perinatal outcomes

**DOI:** 10.3389/fpain.2024.1405465

**Published:** 2024-07-25

**Authors:** Mariano Lanna, Daniela Casati, Chiara Bianchi, Stefano Faiola, Arianna Laoreti, Francesco Cavigioli, Valeria Savasi, Gianluca Lista

**Affiliations:** ^1^ Fetal Therapy Unit “U. Nicolini”, Buzzi Children’s Hospital, University of Milan, Milan, Italy; ^2^ Department of Woman, Mother and Neonate, Buzzi Children’s Hospital, University of Milan, Milan, Italy; ^3^ ASST Brianza, Ospedale PIO X Desio, Milan, Italy; ^4^ Neonatal Intensive Care Unit, Buzzi Children’s Hospital, University of Milan, Milan, Italy

**Keywords:** intrauterine transfusion, fetal analgesia, fetal anemia, alloimmunization, parvovirus B19, cytomegalovirus, chorioangioma, monochorionic twin

## Abstract

**Introduction:**

Intrauterine transfusion is the treatment for fetal anemia resulting from maternal alloimmunization, infections (parvovirus B19 and cytomegalovirus), single demise of a monochorionic twin, chorioangioma, and other rare conditions. Fetal analgesia is mandatory to reduce movement and pain perception during the procedure. This study aims to evaluate perinatal outcomes for such procedures, following the routine use of fetal analgesia in our clinical practice.

**Materials and methods:**

Retrospective analysis of cases from 2009 to 2022, including all confirmed fetal anemia with fetal blood sampling. After fetal analgesia, Rh-negative concentrated red blood cells were transfused, with ultrasonographic follow-up 24 h and 1 week later. In case of suspected brain lesion, magnetic resonance imaging was performed. Elective delivery was considered in case of persistent anemia after 34 weeks. Post-natal follow-up and comprehensive obstetric and perinatal outcomes data were collected.

**Results:**

Altogether 59 anemic fetuses were included, with 34 (57.6%) being hydropic. The causes of anemia were maternal alloimmunization (22, 37.3%), infections (13, 22%), monochorionicity (10, 16.9%), rare conditions (9, 15.3%), and two chorioangiomas (3.4%). The median gestational age at the procedure was 25.2 weeks (18–32 weeks), with no related preterm premature rupture of membranes (<48 h), or side effects from fetal analgesia. Gestational age at delivery was 33 weeks (26–41 weeks), with survival rate of 90%. There were four fetal demises, two termination of pregnancies, and eight neonatal deaths due to persistent severe anemia after preterm delivery. The main contributors to adverse outcome were the type of anemia, and the management with a preterm delivery.

**Conclusion:**

Intrauterine transfusion of red blood cells under analgesia is safe, with low incidence of obstetric complication.

## Introduction

Fetal anemia was the initial focus of investigation for perinatologists, following Liley demonstration of a correlation between bilirubin concentration in amniotic fluid and disease severity (1). Various methods for intrauterine transfusion (IUT) have been utilized (2) and in recent years the procedure has become standard practice in fetal therapy centers worldwide. Donor blood is injected into the umbilical vein, either on the placental side or at the intrahepatic insertion, the latter being our preferred approach to reducing bleeding risk after needle removal (3). Fetal analgesia is essential to minimize movement and pain perception during the procedure (4). The aim of our study was to assess perinatal outcomes in pregnancies undergoing IUT following the routine use of fetal analgesia in our center.

## Methods

A retrospective analysis was conducted on consecutive IUT cases performed at Fetal Therapy Unit “U Nicolini” of Buzzi Children's Hospital in Milan, Italy, from 2009 to 2022, treating fetal anemia from 18 to 34 weeks of gestation. All cases underwent ultrasonographic (US) evaluation with Doppler velocimetry on middle cerebral artery (MCA) to assess peak systolic velocity (PVS), indicating high risk for anemia if values exceeded 1.5 multiple of median (MoM) from reference charts (5).

Other indicators of anemia severity, such as hydrops and signs of cardiac overload, were also considered. Maternal Rhesus antibodies and infections were tested to determine if fetal anemia originated from maternal alloimmunization or infections such as parvovirus B19 (P-B19) and cytomegalovirus (CMV), which were always confirmed on amniotic fluid and fetal blood. Cases of single survivors after intrauterine death (IUD) of a monochorionic (MC) twin, chorioangioma (CA), and any other rare diseases were also included.

After establishing fetal analgesia with fentanyl, intramuscular mivacurium was used to decrease fetal movements. All medications were dosed on estimated fetal weight (6). Fetal blood sampling (FBS) and intrauterine red blood cell (RBC) transfusion were done with a 18 G needle under high resolution US guidance from the umbilical vein; either at the intrahepatic portion or placenta (6). The amount of Rh-negative concentrated RBCs needed for transfusion was calculated based on fetal hematocrit to achieve a fetal hemoglobin level of 14 g/dl (7). The procedure concluded after confirming anemia correction with subsequent blood sampling, usually followed by an MCA-PVS measurement and a 24-h post-procedure ultrasound evaluation to determine discharge and follow-up timing. Repeat IUTs were performed if recurrent anemia was suspected, while magnetic resonance imaging (MRI) was indicated for suspected brain lesions. Elective delivery was an option for early anemia recurrence, or after 34 weeks of gestation. Data on obstetrical complication, such as preterm premature rupture of membrane (pPROM), fetal demise, miscarriage, termination of pregnancy, and preterm delivery (PD), and on perinatal outcome, such as gestational age (GA) at delivery, birthweight, neonatal death (NND), and acquired brain lesions, were collected.

The ethical committee approved the retrospective study (*n* 612/2023), and informed consent for the use of their data was obtained from all patients.

### Statistical analysis

Continuous variables are presented as mean ± SD or range, while categorical variables are expressed as numbers and percentages. The Kruskal–Wallis test compared multiple continuous variables, while chi-square and Fisher's exact tests compared categorical variables (*p* < 0.05 was considered significant). SPSS Statistics v28.0 performed the analysis.

## Results

During the study period, 59 cases were included. Maternal characteristics are summarized in Table 1. The primary cause of fetal anemia was maternal alloimmunization (22, 37.3%), followed by infections, such as cytomegalovirus and parvovirus B19 (13, 22%), single demise in MC pregnancies (10, 16.9%), rare conditions (9, 15.3%), and two cases of chorioangioma (3.4%).

**Table 1:** Characteristics of the population.

Characteristic	59 cases
Maternal age (years, range)	33 (21–45)
Nullipara, *n* (%)	25 (42.3)
ART, *n* (%)	4 (6.8)
Previous procedure for PD, *n* (%)	13 (22)
Poor obstetrical history, *n* (%)^a^	8 (13.5)
Hydrops at diagnosis	34 (57.6)
FGR	8 (13.5)
Cardiac overload^b^	37 (62.7)

ART, assisted reproduction technology; PD, prenatal diagnosis; FGR, fetal growth restriction.

^a^ Previous fetal or neonatal death.

^b^ Presence of moderate or severe tricuspid valve insufficiency or cardiomegaly.

The intrahepatic portion of the umbilical vein was the preferred insertion site for the needle in 53 cases (89.8%), with a median GA at first procedure of 25.2 weeks (18–32 weeks). A total of 97 IUTs were performed, with a range of one to five per pregnancy.

No complications were reported after fetal analgesia, which effectively reduced fetal movements within 2 min of administration.

Complication analysis based on the indication for IUT is detailed in Table 2.

**Table 2:** Comparison of the outcomes between causes of anemia.

	Total (*n* = 59)	Alloimmunization (*n* = 21)	Infections (*n* = 16)	Single MC demise (*n* = 10)	Rare anemias (*n* = 10)	CA (*n* = 2)	*p*
GA at procedure (weeks), median (range)	25.2 (18–32)	27.2 (18–32)	23.3 (20–29)	23.0 (18–27)	26.1 (20–29)	27.4 (25–29)	*.001*
N of IUT (range *n*/case)	97 (1–5)	47 (1–5)	20 (1–3)	10 (1)	18 (1–5)	2 (1)	*NS*
IUD <48 h, *n* (%)	4 (7)	0 (0)	2 (12)	1 (10)	1 (10)	0 (0)	*NS*
TOP, *n* (%)	2 (3)	0 (0)	0 (0)	2 (20)	0 (0)	0 (0)	*NS*
pPROM <48 h, *n* (%)	0 (0)	0 (0)	0 (0)	0 (0)	0 (0)	0 (0)	*NS*
PD <32 weeks, *n* (%)	17 (30)	7 (33)	2 (12)	3 (30)	4 (40)	2 (100)	*.001*
GA at delivery (weeks, range)	33.0 (26–41)	31.9 (26–37)	36.5 (31–41)	32.9 (28–41)	30.8 (29–37)	28 (26–30)	*.001*
Livebirth	53 (90)	21 (100)	14 (87)	7 (70)	9 (90)	2 (100)	*.001*
Birthweight (g, range)	2,227 (700–4,100)	2,038 (700–2,800)	2,490 (1,300–3,700)	2,409 (1,800–4,100)	2,085 (1,000–3,200)	1,350 (900–1,800)	*NS*
NND	5 (8)	2 (9)	0 (0)	0 (0)	2 (20)	1 (50)	*.001*

No cases of pPROM occurred within 48 h following the procedure. However, delivery before 32 weeks occurred in 17 cases (30%), with seven in the alloimmunization group, where preterm delivery was considered due to ongoing management.

Four fetal demises (7%) occurred within 48 h following the procedure, along with two terminations of pregnancies (TOP) for acquired brain injury, and five neonatal deaths (8%) due to persistent severe anemia, resulting in a 90% survival rate (see Table 3 for case descriptions).

**Table 3:** Description of cases with fetal and neonatal demise.

Case		Cause of anemia	GA	Cause of death
1	IUD	Single survivor	23	TTTS
2	IUD	Parvovirus B19	21	Intrahepatic hemorrhage for severe thrombocytopenia
3	IUD	CMV	21	Bleeding from the puncture site for severe thrombocytopenia
4	IUD	Hemochromatosis	20	Bleeding from the puncture site for severe thrombocytopenia
5	TOP	Single survivor	28	Brain injury
6	TOP	Single survivor	20	Brain injury
7	NND	Alloimmune	29	Delivery 1 day after first IUT, technical limitation for a second procedure, severe anemia at birth
8	NND	Alloimmune	26	Delivery for PPROM and labor after one IUT, severe anemia at birth, birthweight 700 g
9	NND	Rare anemia	33	Delivery after one IUT, personal limitation of the patient to return for a second procedure, severe anemia at birth unresponsive to treatment, birthweight 1,865 g
10	NND	Rare anemia	29	Delivery for persistence of fetal anemia after two IUTs, severe anemia at birth unresponsive to treatment, birthweight 1,600 g
11	NND	Chorioangioma	26	Delivery for persistence of anemia and unsuccessful treatment of chorioangioma, severe anemia at birth, birthweight 900 g

TTTS, twin–twin transfusion syndrome.

## Discussion

Our study contributes to assessing the performance of our center in IUT procedures, an audit suggested for any fetal therapy center (8). It confirms the safety of IUT with a moderate risk of fetal (7%) and neonatal (8%) demise and a consistent risk of preterm delivery before 32 weeks (30%). The strength of our study lies in the comparative analysis of IUT indications, including conditions underreported in the literature.

In cases of alloimmune anemia, no fetal demises occurred following the procedure, with only two neonatal deaths due to severe anemia at birth alongside with prematurity, resulting in a 91% survival rate, consistent with literature findings (9), with an 89% survival rate reported.

For infections, fetal demise occurred due to disease-related complications, resulting in an 86% survival rate, similar to other reports (10, 11).

In single survived monochorionic twins, although it has been described that IUT might not prevent adverse neurological outcomes (12, 13), a rescue transfusion was considered to increase survival chances.

For other conditions, due to the small number of cases and the rarity of etiology, it is difficult to perform an analysis on the single variable, even though the persistence of untreatable anemia after birth was the common cause of neonatal deaths.

What is remarkable in the alloimmunization group is the high percentage of premature delivery (7/21, 33%), mostly as a consequence of medical indication by operators unable to perform another IUT in the referral hospital. For many pregnant women living far from our center in our country, it has been difficult to come back while at advanced GA. This is an issue whose solution has been investigated also for other fetal interventions, especially in large countries with a low density of fetal therapy centers (14).

The same problem causes a lack of a long-term neurological follow-up, which is the main limitation of our study.

For rare anemias, the introduction in clinical practice of whole exome sequencing, which was not routinely available at the time of the study period, might help in defining the diagnosis (15), and assessing the correct treatment.

As already reported (16), the introduction of fetal analgesia, the transfusion performed in the intrahepatic portion of the umbilical vein, and the centralization of cases, help achieve better outcomes. Transfusions performed before the onset of a hydrops by operators with a specific expertise (a minimum number of annual procedures of 10, which is the mean of procedures performed at our center) are related to better outcomes.

Fetal analgesia has been validated as a support for *in utero* therapy procedures. It is necessary both to reduce fetal movements, thereby shortening the procedure time and making it easier, and to diminish the perception of pain, which has been reported by various sources to be present from 25 weeks of gestation (6). Research on fetal physiology has determined that nociceptive components of the stress response are present by 19 weeks of gestation, while the perception of pain does not significantly develop until 24–30 weeks of gestation (6). Although further studies are needed to confirm fetal pain perception and determine the gestational age at which it occurs, it seems appropriate to use fetal analgesia in its various available forms starting from 20 weeks. No significant side effects have been described that would contraindicate its use.

In the largest series described by a single center (16), fetal paralysis was negatively associated with procedure complications, and no side effects were described with fetal analgesia.

Intrauterine transfusion is considered a procedure where there is no need for fetal analgesia when performed at the placental insertion of the umbilical vein. Our routine practice is to prefer the intrahepatic portion of the umbilical vein as the injection point to reduce procedure complications (3), so our standard of care for IUT always includes fetal analgesia.

## Conclusion

Intrauterine transfusion of blood products is a safe procedure, with low incidence of obstetric complication. Higher survival rates and better post-natal outcomes were observed in immune anemic fetuses compared with other medical conditions.

## Data Availability

The raw data supporting the conclusions of this article will be made available by the authors, without undue reservation.
